# INFLUENCE OF TOBACCO, ALCOHOL AND DIABETES ON THE COLLAGEN OF CREMASTER MUSCLE IN PATIENTS WITH INGUINAL HERNIAS

**DOI:** 10.1590/0102-6720201600040002

**Published:** 2016

**Authors:** Sérgio Ferreira MÓDENA, Eduardo José CALDEIRA, Marco Antonio O PERES, Nelson Adami ANDREOLLO

**Affiliations:** 1 Department of Surgery, Faculty of Medicine of Jundiaí, Jundiaí; SP, Brazil; 2 Department of Morphology and Basic Pathology, Faculty of Medicine of Jundiaí, Jundiaí; SP, Brazil; 3 Department of Surgery, School of Medical Sciences, State University of Campinas, UNICAMP, Campinas, SP, Brazil

**Keywords:** Muscle cremaster, Collagen, Inguinal hérnia, Alcoholic beverages, Smoking, Diabetes

## Abstract

**Background:**

New findings point out that the mechanism of formation of the hernias can be related to the collagenous tissues, under activity of aggressive agents such as the tobacco, alcohol and diabetes.

**Aim:**

To analyze the collagen present in the cremaster muscle in patients with inguinal hernias, focusing the effect of tobacco, alcohol, and diabetes.

**Methods:**

Fifteen patients with inguinal hernia divided in three groups were studied: group I (n=5) was control; group II (n=5) were smokers and/or drinkers; and group III (n=5) had diabetes mellitus. All subjects were underwent to surgical repair of the inguinal hernias obeying the same pre, intra and postoperative conditions. During surgery, samples of the cremaster muscle were collected for analysis in polarized light microscopy, collagen morphometry and protein.

**Results:**

The area occupied by the connective tissue was higher in groups II and III (p<0.05). The collagen tissue occupied the majority of the samples analyzed in comparison to the area occupied by muscle cells. The content of total protein was higher in groups II and III compared to the control group (p<0.05).

**Conclusion:**

The tobacco, alcohol and diabetes cause a remodel the cremaster muscle, leading to a loss of support or structural change in this region, which may enhance the occurrences and damage related to inguinal hernias.

## RESUMO

**Racional:**

Estudos recentes sinalizam que o mecanismo de formação das hérnias pode estar relacionado aos tecidos colagenosos, sob a ação de agentes agressores como o tabaco, o álcool e o diabete.

**Objetivo:**

Avaliar o colágeno presente no músculo cremaster em pacientes com hérnias inguinais enfocando o efeito do tabaco, álcool e diabete.

**Métodos:**

Foram estudados 15 pacientes com hérnias inguinais divididos em: grupo I (n=5) controles; grupo II (n=5) indivíduos fumantes e/ou etilistas; e grupo III (n=5) indivíduos que apresentavam diabete melito. Todos foram submetidos à correção cirúrgica das hérnias inguinais obedecendo às mesmas condições pré, intra e pós-operatórias. Durante o procedimento cirúrgico, amostras do músculo cremaster foram coletadas para análises em microscopia de luz polarizada, morfometria do colágeno e de proteínas.

**Resultados:**

A área ocupada por tecido conjuntivo foi maior nos grupos II e III (p<0,05). O tecido colágeno ocupou a maior parte das amostras analisadas, em comparação à área ocupada pelas células musculares. O conteúdo de proteínas totais foi maior nos grupos II e III, quando comparado com o grupo controle (p<0,05).

**Conclusão:**

O tabaco, o álcool e o diabete ocasionam remodelação no músculo cremaster, levando à perda de suporte ou alteração estrutural nesta região, podendo intensificar as ocorrências e os danos relacionados às hérnias inguinais.

## INTRODUCTION

The excessive use of alcohol is related to social problems, accidents, trauma injuries, pain, suffering and mortality, inflicting a harsh economic sanction to society and currently becoming a public health problem^32^. Besides alcohol, the smoke addiction has raised particularly among the youth and it is responsible for four million of deaths yearly in the world, and this is due to cultural reasons, easy access, familiar influence and industrial marketing. In USA, for example, every day 2.200 young people smoke cigarettes for the first time, and at about 830 became regular users^29^. 

More than 4.000 toxic substances can be isolated from the cigarettes, and the nicotine is the responsible for the addictiveness. Furthermore, the cigarettes´ effect may be enhanced by the use of alcohol, easing the occurrence of precancerous and cancerous lesions ^29^. Other several problems, also related to the tobacco use, in tissues of the digestive system are described^23^ ^,^ ^27^ ^,^ ^30^. Clinical and experimental trials indicate the toxic effects of these substances, which may cause changes in the skin and in the subcutaneous tissue, in the pH of the urine, vascular disorders, and vasospasm, in addition to inflammatory processes^6^ ^,^ ^8^ ^,^ ^27^.

The inguinal region is anatomically characterized by the inguinal canal, the most common region of occurrence of inguinal hernias. The anterior wall, its ceiling, posterior wall and floor it are constituted, respectively, by the aponeurosis of the external oblique muscle of the abdomen, the internal oblique and transversus abdomen muscles, the fascia transversalis and parietal peritoneum and the aponeurosis of the external oblique muscle of the abdomen. This channel it is located the spermatic cord surrounded by the main muscle of the region, the cremaster muscle, mainly made up of striated muscles^3^ ^,^ ^10^ ^,^ ^18^ ^,^ ^25^. All the tissues, including the muscle ones, hold a support compartment, which owns blood, lymphatic vessels and nerves, presenting also connective tissues in which the collagen type is the predominant one, even though other types are also present. At normal environments, the collagen is regular among the cells; however, at adverse conditions these components can be increased^12^ ^,^ ^16^ ^,^ ^20^. 

New findings point out that the mechanism of formation of the hernias can be related to the collagenous tissues, under activity of aggressive agents such as the tobacco, alcohol and diabetes. However, there is still the need of quantitative results that can indicate the real alterations of the tissue, mainly in the cremaster muscle^9^ ^,^ ^10^ ^,^ ^13^. Bingöl-Koloğlu et al., when evaluating the changes in the cremaster muscle, could not get enough data that would support the real relationship of these alterations with the hernias' occurrence, thus suggesting the need of further studies about this theme^1^.

The aim of the current study was to evaluate the collagen present in the cremaster muscle, in patients with hernias, relating the occurrence of these lesions with smoking, alcoholism and diabetes. 

## METHODS

### Collection of tissue samples 

Fifteen male and white patients were evaluated and submitted to inguinal hernia repair in the University Hospital of the Faculty of Medicine, in Jundiai, São Paulo, SP, Brazil. They were divided in three groups: group I with five healthy men (control); group II with five smoking and/or drinking ones; and group III with five people that had diabetes mellitus. Table 1 shows the age range and the mass body index alterations in the patients included.

**TABLE 1:** The age range and the mass body index in the study groups

Variables/groups	Group I	Group II	Group III
Minimum age (years)	19	38	54
Maximum age (years)	72	75	81
Age average (years)	38.1	64.5	65
Minimum BMI	19.4	20.6	20.5
Maximum BMI	29.0	28.7	26.3
Average BMI	23.5	25.1	23.8

The research was approved by the Ethical Committee of the Faculty of Medicine in Jundiaí (protocol number 625.555), and all the patients filled the Agreement Term. The participants included in the study were submitted to the same pre, intra and postoperative conditions, and during the hernia repair it were collected samples of the cremaster muscle, with at about 1,5 cm, through dissection with a surgical scalpel blade, taking as a reference the limits of the hernia and the walls of the inguinal canal. The region was photographed with a digital camera for hospital storage. It is important to highlight that possible alterations in the tissues - such as coloring, texture and consistence - were microscopically observed in case they were visible among the groups. 

### Polarized light microscopy 

The samples were collected from each patient, fixed in Bouin (saturated aqueous solution of picric acid - 75 ml formaldehyde - and 25 ml glacial acetic acid - 5 ml) for 12 h for further processing and inclusion in paraffin. For the inclusion in paraffin, the tissues were washed in alcohol 70% and, afterwards, were dehydrated in a raising series of alcohols (alcohol 80% - 2 times, absolute alcohol - 3 times; 1 to 2 h each). Then, the fragments were cleared in xylene for 1 to 2 h until they become translucent and after were embedded in paraffin and plastic polymers (Paraplast Plus) to 56^0^ C for about 1 h and, afterwards, were carefully positioned on the bottom of plastic forms so that transverse histological sections could be obtained. The blocks were formed for the obtainment of flat surfaces and then were sectioned with five micrometers thick. Finally, the fragments were placed on albumin-blades and taken to an oven at 60^0^ C. After preparation of the cuts, they were stained through the histochemical Picrosirius method (saturated aqueous picric acid added with 0.1 g of red Syrian F3B, Sirius Red F3B-Bayer) for evaluation of the fibrillary components of the extracellular matrix^19^. The findings were photographed under a light microscope Nikon Eclipse E100 for microscope with polarized lens system, together with the SD-CCD image acquisition system 3.3, from the Department of Morphology and Basic Pathology from the same faculty. All the observations employed objective 40x.

### Morphometric of the collagenous components 

The fragments chosen for polarized light microscopy were used for the quantification of the collagenous components. The volume density of these components were calculated by the average of four regions of each histological cut, through the following equation: 



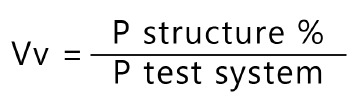



Was considered P structure as the number of points over the studied structure and P test system as the total number of points of the reticle integration quadrilateral used. Afterwards, it was performed the measuring of the total relative area occupied by the muscle fibers and by the collagenous components through the image analysis system Image J 1.39 (Image Processing and Analysis in Java, National Institute of Health, Maryland, USA)^35^.

### Biochemical analysis 

Other fragments of the collected tissues were stored, split (80° C) and homogenized in an extraction buffer (150 mM sodium chloride, 1% NP-40 or Triton X-100, 0.5% sodium deoxycholate, 0.1% SDS, 50 mM Tris, pH 8.0) at 4° C in "Polytron PTA 20S generator" (Brinkmann Instruments EN 10/35 mode) at full speed for 30 s. The homogeneous tissue was centrifuged at 11000 rpm for 30 min for the removal of insoluble material. The total concentration of protein contained in the supernatant was determined through the Bradfort method^4^.

### Statistical analysis

The statistical study was performed for the following variables: relative area of muscle fibers (%), relative area and volume density of collagenous fibers (%), and total level of proteins (mg/dl) through the technic of variables analysis (ANOVA), complementing the non-parametric test of Kruskal-Wallis in 5% level of significance. 

## RESULTS

During the surgical procedure all the tissues were evaluated regarding coloration, texture and consistence, and macroscopically it was not observed any relevant difference among the collected samples. 

### Biochemical analysis 

The analysis of the content of total proteins in the samples of the cremasteric tissue indicated that there was a relevant increase in the groups II and III when compared with group I, at significance level p<0,05 (Table 2).

**TABLE 2:** The protein levels (mg/dl) among the studied groups

	Total proteins (mg/dl)
Group I (Control)	2.17±3.12^a^
Group II	9.58±5.78^b,d^
Group III	10.36±6.26^c,d^

Values expressed through the average±standard deviation; ^a ,b,c^ they differ at 1% of significance (p≤0.01); ^d^ they do not differ at 5% of significance (p≥0.05)

### Microscopy of polarized light 

**FIGURE 1:**
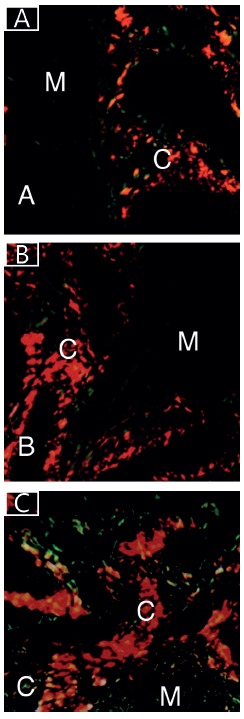
Photomicrography of the cremaster muscle in the patients of the groups I, II e III: A) in group I, it is observed in the Sirius Red coloration the area occupied by the connective tissue (C) and the black area occupied by the muscle cells (M); B) in the group II it is noted the area occupied by the connective tissue (C) and the black area occupied by the muscle cells (M); C) in group III it is observed the area occupied by the connective issue (C) and the black area occupied by the muscle cells (M) (Picrosirius Red - 300x).

In the group I (control) a predominance of muscle cells was observed. However, in the groups II and III, it was noted a predominance of the area occupied by the connective tissue and a reduction of the area occupied by the muscle cells, observing a statistically significant difference (p< 0.05, Figure 1 and Table 3).

**TABLE 3:** The relative area occupied by the muscle cells (%) and by the connective tissue (%) according to the studied groups

	Muscle cells (%)	Connective tissue (%)
Group I	89.23±03.38^a^	11.77±02.98^a^
Group II	43.20±14.56^b,d^	57.80±29.96^b,e^
Group III	39.26±15.37^c,d^	61.74±31.87^c,e^

Values expressed through the mean±standard deviation; ^a,b,c^ they differ at 1% of significance (p≤0.01); ^d^ they do not differ at 5% of significance (p≥0.05); ^e^they do not differ at 5% of significance (p≥0.05)

## DISCUSSION

The connective tissue is composed by three groups of extracellular proteins: the proteoglycans, the glycoproteins and the collagens. The proteoglycans have functions in regulating the structure and permeability of the tissue. The glycoproteins act in the cell-cell interactions. The collagens are the main responsible for the matrix structure and support of the connective tissue. Among vertebrates and invertebrates there are about 28 known types of collagens, designated by Roman numbers (I - XXVIII), in chronological order of their discovery ^22^. 

The amount of collagen varies according to the tissue, and the collagen type I is present mainly in the structure of the tendons, ligaments and striated muscle fibers^22^.

Clinical and experimental trials that employ the morphologic techniques have been showing that different factors may compromise both the connective tissue as well as its support storage, promoting intense morphological and functional damage^20^ ^,^ ^22^. Therefore, morphological quantitative analysis of the collagenous tissues of the cremaster muscle are necessary and can particularly contribute for the comprehension of the effects of the tobacco, alcohol and the own diabetes in the occurrence of hernias in the inguinal region. 

The polarization microscopy has been traditionally employed as an important tool in the study of these fibrillary structures, among other functions. Schmidt (1924)^31^ was one of the first authors to apply the polarization microscopy in studies of biological structures and, since then, several researches emphasize the application of such technique with the aim of morphologically observe biological materials and obtain important responses about these structures^24^ ^,^ ^28^. 

The diabetes mellitus, besides the tobacco and the alcohol, can also be related with structural alterations in different tissues, indeed compromising their functions^6^ ^,^ ^8^ ^,^ ^30^. In this sense, some studies relate that patients with hernias in the abdomen wall also have diabetes, associated or not with the use of tobacco or alcohol. However, these are studies that only focus these elements as being risk factors for the occurrence of hernias, but do not explain which changes happen in the tissues^12^ ^,^ ^16^.

The muscle tissues, as other ones, present a support storage that is essential for the cellular homeostasis. At these storages there are the connective tissues that at normal conditions present themselves among the cells, thus structuring the organ´s construction, as well as providing subsidies for its maintenance^12^ ^,^ ^21^ ^,^ ^22^ ^,^ ^26^ ^,^ ^33^.

Smoking and alcohol consumption causes a lot of damage to humans, which predisposes users to different diseases; however, the literature does not yet holds background and data enough in order to confirm how the exposition to these agents can really be related to cells´ alterations at different tissues, fact which suggests that more studies need to be performed ^8^ ^,^ ^27^ ^,^ ^30^. 

The diabetes mellitus is also associated with structural alterations in different organs and tissues, indeed compromising its functions^5^ ^,^ ^7^. Some authors report that patients with hernia of the abdomen wall have diabetes at 8.4% of the cases and describe that 43% smoke and 7.4% are alcoholic. Furthermore, they relate that smokers have alterations in the metabolism of the inguinal tissues, thus contributing for a higher occurrence of such type of hernia^3^ ^,^ ^5^ ^,^ ^13^.

The occurrences of other diseases and their association with collagen variations have also been described in literature. Bonduki et al., studied 15 patients with infertility symptoms and related the collagen present in the uterine muscles with the tumors in the region^2^. Wang et al., studying patients with hepatic fibrosis, also observed that in the majority of the cases there are alterations in the fibrillary components of this organ^34^. On the other hand, Gudiene et al., evaluating the diameter of blood vessels, showed that the age of people may alter the tissue collagen^15^.

Although the patients included in this study had varying ages, they were all male, white and with a very similar body mass index. The results indicated, in patients considered healthy and with inguinal hernias, a larger area occupied by the muscle cells and a smaller one formed by the connective tissue. On the flip side, in individuals of the groups II and III, made up of smokers, drinkers or diabetic people, the majority of the cremaster muscle samples showed intense accumulation of connective tissue.

The quantification of the total proteins in these tissues, through the biochemical analysis, were similar to the results obtained in the polarized light microscopy, also showing a significant increase of these levels in the samples obtained in participants from groups II and III, who had a relation with smoking, alcoholism or diabetes. 

These results were considered homogeneous; therefore, the aggressive agents may promote an increase in the area occupied by collagenous storage and may thus alter the structure and function of the tissue. These findings confirm the relationship of the hernias with the damaging agents and are in the agreement with the literature. Wang et al. reported that the most intense changes in the collagen metabolism were found in the anterior sheath of the rectus abdominis muscle of patients with direct inguinal hernia, when compared to patients with indirect inguinal hernia or the ones in the control group, with no statistically significant difference. However, the authors did not mention the age of the patients nor any association with diabetes, alcoholism or tobacco^33^.

Fachinelli and Maciel Trindade, when studying patients with epigastric, umbilical and incisional hernias, discovered that the highest the hernias` incidence, the smaller amount of collagenous tissue the patients had in these areas^11^.

Gonçalves et al., analyzed samples of the transversalis fascia and the anterior sheath of the rectus abdominis muscle of 40 men with inguinal hernia type II and IIIA of Nyhus, comparing them with 10 control fresh cadavers, with the same age, without hernia, using the immunohistochemistry studies of collagen I, collagen III and elastic fibers. The research concluded that the amount of fibrillar components of the extracellular matrix was not alterated in patients with and without inguinal hernia^14^. 

Henriksen et al., evaluated patients with three different types of hernias: unilateral inguinal hernia, multiple hernias (more than three types) and incisional hernias. Patients without hernias submitted to cholecystectomy served as a control group. The authors analyzed the collagen type I, III, IV and V and concluded that patients with hernias presented altered collagen metabolism, and that the study of the turnover of type IV collagen is able to predict the presence of inguinal and incisional hernias. Therefore, the regulation of the turnover of type IV collagen may be a crucial factor for the occurrence of hernias^16^. The same authors, in a recent systematic literature review published, analyzed a total of 55 original articles, evaluating connective tissue alterations in patients with abdominal wall hernias. They concluded that patients with inguinal and incisional hernias exhibit a decreased type I to III collagen ratio in fascia and skin biopsies with the most pronounced alterations found in patients with direct inguinal hernia and hernia recurrence ^17^.

Concluding, all these findings show the complexity that involves the formation of hernias and their relationship to the abdominal wall. It is important to highlight that this research it was not approached the severity of hernias injuries, nor was evaluated postoperative course of studied cases, being these issues possible aims of new researches. It is important to emphasize that the quantification of the tissue activity may contribute to the comprehension of the formation and incidence of the hernias, complementing the results obtained up until now. 

## CONCLUSIONS

Tobacco and alcohol, together with diabetes mellitus, cause a re-modeling in the cremaster muscle, leading to a loss of support or structural alteration in this region, being able to intensify the occurrences and damages related to the inguinal hernias. 
